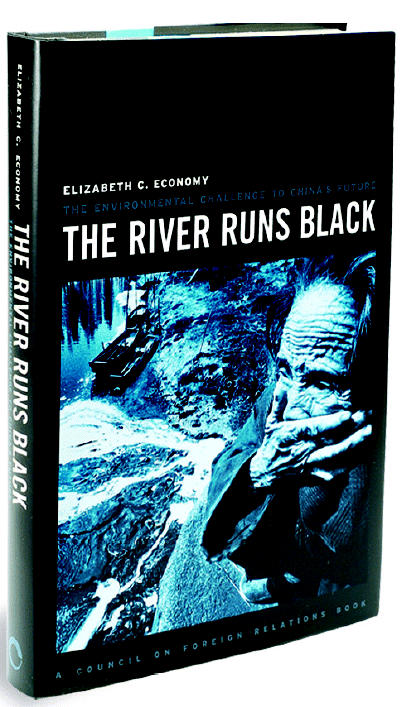# The River Runs Black: The Environmental Challenge to China’s Future

**Published:** 2004-12

**Authors:** Giovanna Dore

**Affiliations:** Giovanna Dore is an environmental specialist with the Environment and Social Development Unit of the East Asia and Pacific Region at the World Bank. Her sectoral expertise lies in institutional development and economics for environment and natural resources management for China, Indonesia, Mongolia, the Philippines, and Vietnam.

By Elizabeth C. Economy

Ithaca, NY:Cornell University Press, 2004. 337 pp. ISBN: 0-8014-4220-6. $29.95 cloth.

In *The River Runs Black*, Elizabeth C. Economy strikes a fine balance between acknowledging the merits of the country’s continued economic transformation (especially in terms of reduced social, economic, and human cost of poverty), presenting an overview of the environmental challenges and impacts of such sustained economic growth, and how the range and significance of these challenges have changed and increased, particularly over the last three decades. Drawing on various original and secondary sources, she analyzes how these challenges are affecting the environmental agenda, identifies environmental management strategies and priorities, proposes lessons from the international experience, and suggests a course for improving environmental quality in China over the short to medium term.

Among the highlights of the book are the first five chapters, which, thanks to a dynamic and lean structure, take the reader through several centuries of Chinese history and environmental tradition. In Chapter 2, “A Legacy of Exploitation,” a sharp overview of how the traditional concepts and institutions of Confucianism have played a formidable role in shaping China’s original nonenvironmental development policies is complemented by discussion of the reasons why the relatively more eco-friendly philosophies of Taoism and Buddhism had a limited impact in the consciousness of Chinese leaders and people. From the historical and philosophical excursus, the author concludes that the predominance of Confucianism produced a long tradition of exploiting the environment for human needs, with little or no concern for the long-term impacts of the practical application of this philosophy—deforestation and exploitation of mining resources, poor water resources management, and, more important, no formal administrative structure in place to manage and protect environment and natural resources, leaving such tasks to the country’s leaders. Chapter 5, “The New Politics of the Environment,” the most interesting of these five chapters (and possibly of the entire book), offers many insights on the workings of civil society and nongovernmantal organizations’ participation in environmental decision making, their ability to influence it given existing policy and political constraints, and their limited access to funding and reliable information about environment and natural resources management.

Unfortunately, the second part of the book does not live up to this quality. The information of the last three chapters is less original and especially lacks most of the intriguing introspection and analysis offered earlier in the book. Chapter 7, “Lessons from Abroad,” offers a brief, detailed account of how other countries have dealt with environmental challenges in circumstances similar to those of China and how the international community has helped. This chapter is the most interesting of the last three because of the uncharacteristic scope of several of the comparisons presented (such as those with former Soviet Union and Eastern European countries). Yet the author fails to identify what the experiences of these other countries really mean for China and, especially, how China can best use the lessons learned from those experiences on balancing sound environmental management with economic demand while maintaining social stability.

Despite some reading fatigue from the last three chapters, *The River Runs Black* can please many audiences: those who are knowledgeable about China and the political economy of its environmental problems, and those who are only superficially interested in China and/or in environmental affairs. The book could also be excellent background reading for a graduate program in environmental policy and management and/or on China.

## Figures and Tables

**Figure:**